# Hypothesis-generating network toxicology analysis of potential molecular convergence between a polystyrene oligomer proxy and papillary thyroid carcinoma

**DOI:** 10.3389/fendo.2026.1942165

**Published:** 2026-10-14

**Authors:** Weidong Xie, Jian Tang, Xiaoyu Qian

**Affiliations:** Department of Head and Neck Surgery, The First Hospital of Jiaxing (Affiliated Hospital of Jiaxing University), Jiaxing, China

**Keywords:** machine learning, molecular convergence, network toxicology, papillary thyroid carcinoma, polystyrene oligomer proxy, single-cell transcriptomics

## Abstract

**Background:**

Polystyrene microplastics (PS-MPs) are emerging environmental contaminants with endocrine-disrupting and pro-inflammatory effects, but whether PS-MPs-associated molecular targets converge with papillary thyroid carcinoma (PTC) alterations remains unclear. This hypothesis-generating study aimed to identify candidate molecular networks and cellular contexts shared between a simplified polystyrene computational proxy and PTC.

**Methods:**

Putative targets from CTD and from TargetNet/SwissTargetPrediction using a five-styrene-unit oligomer proxy were intersected with thyroid cancer-related genes from OMIM and GeneCards. Protein functional-association and enrichment analyses were followed by differential expression analysis in GSE33630 (45 normal and 49 PTC samples). LASSO and SVM-RFE identified core genes; stability was examined by repeated nested resampling conditional on the seven-gene candidate panel. Individual-gene tumor-normal discrimination was assessed in GSE33630 and GSE60542. Protein patterns were evaluated qualitatively in the Human Protein Atlas and preliminarily in 15 matched PTC-adjacent tissue pairs. CIBERSORT-estimated immune fractions and two single-cell datasets were analyzed as exploratory context.

**Results:**

Among 144 putative proxy-associated targets and 2,139 thyroid cancer-related genes, 75 were shared. Seven candidates overlapped with 1,306 PTC differentially expressed genes, and BCL2, BAX, and FN1 were selected by both feature-selection methods and remained stable across the tested GeneCards thresholds and CTD-only sensitivity analysis. BCL2 was downregulated, whereas BAX and FN1 were upregulated in PTC; individual-gene AUCs were 0.963, 0.941, and 0.965 in GSE33630 and 0.942, 0.907, and 0.955 in GSE60542. HPA images and clinical IHC supported the same protein-level directions. After multiple-testing correction, only the CIBERSORT-estimated neutrophil fraction differed between groups (higher in PTC; q=0.0021). Single-cell analysis localized the three genes across epithelial, stromal, and immune compartments.

**Conclusion:**

BCL2, BAX, and FN1 are candidate molecular nodes at the convergence of polystyrene-proxy annotations and PTC-associated alterations. The findings do not demonstrate patient PS-MPs exposure, intact-particle effects, or causality, and require validation in material-characterized exposure experiments and exposure-aware clinical studies.

## 1 Introduction

Thyroid cancer is the most common malignancy of the endocrine system, and its global incidence has continued to rise over the past several decades (1, 2). Although the overall prognosis of thyroid cancer is generally favorable, established risk factors, including ionizing radiation, abnormal iodine intake, and genetic susceptibility, account for only a subset of cases (3). Because the thyroid gland is sensitive to exogenous endocrine disruptors, oxidative stress, and chronic inflammation, identifying potential molecular links between emerging environmental pollutants and thyroid cancer is important for understanding otherwise unexplained disease risk.

In recent years, increasing attention has been paid to the potential role of environmental pollutant exposure in thyroid carcinogenesis. Several organic pollutants have been shown to interfere with thyroid hormone synthesis and metabolism (4). Among these pollutants, microplastics (MPs), as an emerging class of environmental contaminants, have become a global public health concern because of their potential effects on human health (5, 6). MPs have been detected in multiple human biological compartments, including blood and placenta (7, 8).

In oncology, accumulating evidence suggests that MPs may be associated with the development or progression of several malignancies, including breast cancer, lung cancer, and colorectal cancer (9–11). Animal studies have further shown that MPs can induce toxic effects such as oxidative stress, inflammatory responses, and DNA damage (12). With respect to the thyroid, zebrafish models have indicated that polystyrene nanoparticles can alter the expression profile of genes related to the thyroid axis, suggesting potential endocrine-disrupting properties (13). However, the relationship between MPs and thyroid cancer remains poorly understood, and uncertainty in human exposure assessment and causal inference remains substantial (46, 47).

Polystyrene (PS), a representative plastic polymer, is widely used in consumer products and industrial applications and is a plastic type of particular concern in food-chain and environmental samples (14). Previous evidence also suggests that PS may interfere with thyroid function (13). Against this background, the present study integrated bioinformatics analyses with clinical sample-based immunohistochemical validation to explore potential molecular convergence between putative polystyrene microplastics (PS-MPs)-associated targets and papillary thyroid carcinoma (PTC)-associated alterations.

## 2 Materials and methods

### 2.1 Collection of putative polystyrene-proxy-associated target genes

Because polystyrene is a polymeric compound, a polystyrene oligomer containing five styrene monomer units was used as a computational proxy for PS-MPs to represent the chemical repeating unit of polystyrene at the computational level (10). The two-dimensional chemical structure and SMILES format of the oligomer were obtained from the PubChem database (https://pubchem.ncbi.nlm.nih.gov). To comprehensively identify putative polystyrene-associated target genes, three commonly used toxicogenomic and target-prediction databases were searched: the Comparative Toxicogenomics Database (CTD; http://ctdbase.org) (15), TargetNet (http://targetnet.scbdd.com) (16), and SwissTargetPrediction (http://www.swisstargetprediction.ch) (17, 18). The oligomer provides a discrete chemical input for target prediction but does not reproduce particle size, morphology, surface chemistry, aging, aggregation, protein-corona formation, additives, adsorbed contaminants, or cellular uptake.

In CTD, “Polystyrenes” was used as the search term, and genes with reported chemical-gene interactions in at least two published studies were retained (19). In TargetNet, predicted targets were retained using a probability threshold of >0.6 and an AUC of ≥0.7 (20). In SwissTargetPrediction, targets with a probability score >0.05 were retained (21). Targets from the three sources were standardized using UniProt-approved gene symbols, merged, and deduplicated to generate the final set of putative polystyrene-associated targets (22). CTD contributed 80 literature-curated genes, TargetNet 48 unique predicted genes, and SwissTargetPrediction 28 predicted genes; because the sources overlapped, their deduplicated union contained 144 genes. Source membership and evidence class are reported in **Supplementary Material 1**, **Supplementary Table S1**. Data from CTD, TargetNet, and SwissTargetPrediction were obtained on January 27, 2026.

### 2.2 Collection of thyroid cancer-related target genes

Thyroid cancer-related target genes were retrieved from Online Mendelian Inheritance in Man (OMIM; https://omim.org) (23) and GeneCards (https://www.genecards.org) (24) using “Thyroid Cancer” as the search term. In GeneCards, the top 2,000 genes ranked by relevance score were retained to prioritize genes most closely associated with the disease while preserving broad disease coverage. Genes obtained from OMIM and GeneCards were standardized by gene symbol, deduplicated, and manually checked before being merged into a thyroid cancer-related candidate gene set. To assess threshold dependence, the intersection was also repeated using the top 500, 1,000, 1,500, and 3,000 GeneCards genes, each combined with OMIM (**Supplementary Material 1**, **Supplementary Tables S2**, **S4**).

### 2.3 Identification of shared targets between PS-MPs and thyroid cancer

The putative polystyrene-associated target set was intersected with the thyroid cancer-related candidate gene set to identify shared targets. The intersection was visualized using a Venn diagram. As a source-restriction sensitivity analysis, the downstream disease-gene and PTC-DEG intersections were repeated using the CTD-curated subset alone (**Supplementary Material 1**, **Supplementary Tables S3**, **S4**).

### 2.4 Construction of the PPI network

To evaluate the functional relationships among shared targets between polystyrene-associated annotations and thyroid cancer at the protein level, a protein-protein interaction (PPI) network was constructed using the STRING database (25). The shared target genes were submitted to STRING with the species restricted to Homo sapiens (species = 9606). The interaction type was set to functional association, and the minimum confidence level was set to high confidence (required_score = 700). After downloading the interaction results, R 4.3.3 and the igraph package were used to calculate topological features, including the number of nodes, interaction edges, connected components, and degree. The PPI network was then visualized. STRING functional associations do not necessarily represent direct physical protein binding.

### 2.5 Functional enrichment analysis

Gene Ontology (GO) and Kyoto Encyclopedia of Genes and Genomes (KEGG) pathway enrichment analyses of the shared target genes were performed using the clusterProfiler package in R (26). GO enrichment analysis included three categories: biological process (BP), cellular component (CC), and molecular function (MF). KEGG pathway analysis was used to identify significantly enriched signaling pathways. The default human annotation backgrounds supplied by org.Hs.eg.db for GO and the human KEGG annotation were used. A Benjamini–Hochberg-adjusted P value < 0.05 was considered statistically significant. Enrichment was interpreted as statistical over-representation rather than evidence of pathway activation.

### 2.6 GEO data acquisition and differential expression analysis

The gene expression dataset GSE33630 was downloaded from the Gene Expression Omnibus (GEO; https://www.ncbi.nlm.nih.gov/geo). This dataset included 45 normal thyroid tissue samples and 49 papillary thyroid carcinoma (PTC) tissue samples. Differential expression analysis was performed using the GEO2R online tool provided by GEO to improve the reproducibility and transparency of the analysis (27). Differentially expressed genes (DEGs) were defined using the criteria of |log2 fold change (log2FC)| > 1 and a false discovery rate (FDR)-adjusted P value < 0.05. DEGs were visualized using volcano plots.

### 2.7 Intersection of DEGs with shared targets

DEGs identified by GEO2R were intersected with the shared targets identified in Section 2.3. This analysis yielded molecular convergence candidates for which polystyrene-associated annotations overlapped PTC-associated expression alterations. The intersection was visualized using a Venn diagram.

### 2.8 Identification of core genes using machine learning

Two machine learning approaches, Least Absolute Shrinkage and Selection Operator (LASSO) regression and Support Vector Machine-Recursive Feature Elimination (SVM-RFE), were used to identify core genes. LASSO regression was performed using the glmnet package in R (28) for binary classification, with family set to binomial and alpha = 1. Ten-fold cross-validation and lambda.min were used to select the penalty. SVM-RFE (29) used a linear-kernel support vector machine, recursive feature ranking, and five-fold cross-validation. Genes shared by the two methods were defined as the final core genes. Feature-selection stability was additionally assessed by stratified five-fold outer resampling repeated 10 times (50 held-out folds), conditional on the fixed seven-gene candidate panel. Within each outer training set, scaling, L1 regularization, SVM-RFE feature number, and SVM tuning were selected in inner five-fold loops; the corresponding outer test fold was not used for these steps.

### 2.9 Evaluation of core gene expression differences and tumor-normal transcriptomic discrimination

For the core genes jointly identified by LASSO and SVM-RFE, expression differences between normal thyroid tissues and PTC tissues were further assessed in the GSE33630 expression matrix. Receiver operating characteristic (ROC) curves were generated using the pROC package in R (30), and the area under the curve (AUC) was calculated to describe tumor-normal transcriptomic discrimination. For independent assessment, GSE60542, comprising 33 PTC samples and 30 control samples, was used as an independent cohort to assess expression direction and individual-gene ROC performance.

### 2.10 Validation using the human protein atlas

The Human Protein Atlas (HPA; https://www.proteinatlas.org/) (31) was used to assess qualitatively whether the histological protein-expression patterns of the core genes were consistent with the transcriptomic findings. For each protein, normal-thyroid and PTC panels were selected using the same antibody but originated from different public patients; tissue category, patient identifier, antibody identifier, and the qualitative selection criteria are reported in **Supplementary Material 1**, **Supplementary Table S22**.

### 2.11 Immunohistochemical validation using clinical samples

To validate the expression of the core genes at the tissue protein level, immunohistochemical (IHC) staining was performed on archived, 4% paraformaldehyde-fixed, paraffin-embedded tissue sections. Fifteen postoperative cases with pathologically confirmed PTC and available matched tissue were selected retrospectively using a random-number generator. Subacute thyroiditis and other non-Hashimoto inflammatory thyroid diseases were excluded; Hashimoto thyroiditis was not excluded and was recorded for each patient. Matched adjacent non-tumor tissue was obtained at least 2 cm from the tumor, was grossly free of an evident lesion, and was histologically confirmed to be tumor-free. The cohort included 9 women and 6 men (mean age, 49.0 ± 11.9 years); AJCC eighth-edition categories and de-identified patient characteristics are reported in **Supplementary Material 1**, **Supplementary Table S23**. This study was approved by the Ethics Committee of Jiaxing First Hospital (2026-LP-410). After deparaffinization and rehydration, sections underwent Tris-EDTA antigen retrieval (pH 9.0), endogenous-peroxidase blocking, and 3% BSA blocking.

The sections were incubated overnight at 4 °C with antibodies against BCL2 (Proteintech, 26593-1-AP, 1:400), BAX (Abcam, ab32503, 1:200), and FN1 (Proteintech, 15613-1-AP, 1:400). The sections were then incubated with an HRP-conjugated POLY-HRP goat anti-rabbit secondary antibody (Hangzhou Haoke Biotechnology Co., Ltd., HKI0026), developed with DAB, and counterstained with hematoxylin. Positive and negative controls were included in each staining run. H-scores across the entire tissue area were quantified in Visiopharm by independent personnel masked to original specimen identifiers and clinicopathological information. Paired H-scores were summarized with mean differences and 95% confidence intervals and assessed using paired t tests and exact Wilcoxon signed-rank tests, with Bonferroni correction across the three markers within each test family. Full staining, masking, control, segmentation, H-score, and QC procedures are provided in **Supplementary Material 2**.

### 2.12 Immune infiltration analysis and correlation between core genes and immune cells

To evaluate changes in the immune microenvironment of PTC tissues, relative fractions of 22 immune cell types were estimated from GSE33630 using CIBERSORT (32), with 1,000 permutations and quantile normalization. Of 94 input samples, 42 met the prespecified CIBERSORT output P < 0.05 criterion: 15 of 45 normal samples and 27 of 49 PTC samples. Differences between groups were assessed using two-sided Wilcoxon rank-sum tests with Benjamini–Hochberg correction across 22 fractions. Spearman correlations between three core genes and 22 estimated fractions were corrected across 66 tests in the pooled 42 samples and were also examined separately in the 27 PTC samples.

### 2.13 Single-cell transcriptomic analysis

To further clarify the potential cellular sources of the core genes in PTC tissues, the publicly available single-cell transcriptomic dataset GSE184362 was included as the primary analysis dataset (33), and GSE191288 was used as a sensitivity analysis dataset (34). GSE184362 comprised 23 samples from 11 patients: 7 primary tumors, 6 adjacent non-tumor tissues, 8 lymph-node metastatic lesions, and 2 subcutaneous metastatic lesions. GSE191288 included six bilateral tumor samples from three patients and one non-tumor sample. Patients and samples, rather than individual cells, were regarded as the biological replicate units.

Single-cell analysis was performed in R 4.3.3 using Seurat 5.5.0 (35). Quality control criteria were nFeature_RNA ≥ 200, nCount_RNA ≥ 500, and percent.mt ≤ 25%. Data were normalized using LogNormalize; 3,000 variable features, 30 principal components, dimensions 1–20 for neighbors and UMAP, and a clustering resolution of 0.5 were used. Cell types were annotated using canonical marker genes. The expression distributions of the core genes were summarized across tissue origins and cell types without cell-level inferential testing.

## 3 Results

### 3.1 Identification of shared targets and molecular convergence candidates

After integration and deduplication of targets from CTD, TargetNet, and SwissTargetPrediction, a total of 144 putative polystyrene-associated target genes were obtained. After merging and deduplicating results from OMIM and GeneCards, 2,139 thyroid cancer-related genes were identified. Intersecting the two gene sets yielded 75 shared targets (**Figure 1A**). The top-500, 1,000, 1,500, 2,000, and 3,000 GeneCards analyses yielded 39, 53, 68, 75, and 84 shared genes, respectively; BCL2, BAX, and FN1 were retained at every threshold. The CTD-only analysis yielded 57 shared genes and retained the same seven PTC-DEG candidates, including the three core genes. Specific gene names, source evidence, and sensitivity results are provided in **Supplementary Material 1**, **Supplementary Tables S1–S4**.

**Figure 1:**
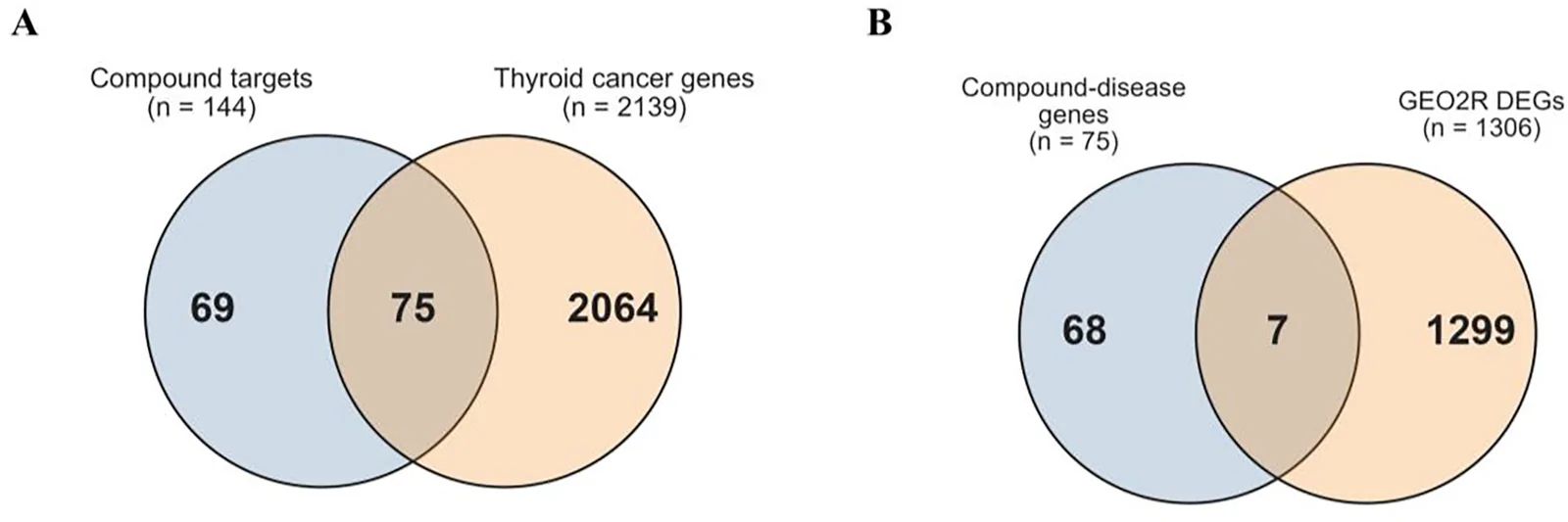
Intersection of putative polystyrene-associated targets, thyroid cancer-related genes, and PTC differentially expressed genes. **(A)** Intersection between putative polystyrene-associated targets and thyroid cancer-related genes. **(B)** Intersection between the shared targets and GEO2R-derived differentially expressed genes.

### 3.2 PPI network analysis of shared targets

STRING analysis showed that the 75 shared targets formed 508 high-confidence functional-association edges. The network contained six connected components, with the largest connected subnetwork comprising 70 nodes, accounting for 93.3% of all shared targets. AREG, CASR, GPX1, ID1, and MKI67 were not included in the main connected network. Node- and edge-level network data are provided in **Supplementary Material 1**, **Supplementary Tables S5**, **S6**.

These findings indicate that most shared targets could be incorporated into a major functional-association network. The topology provided descriptive context for subsequent GO and KEGG enrichment analyses (**Figure 2**).

**Figure 2:**
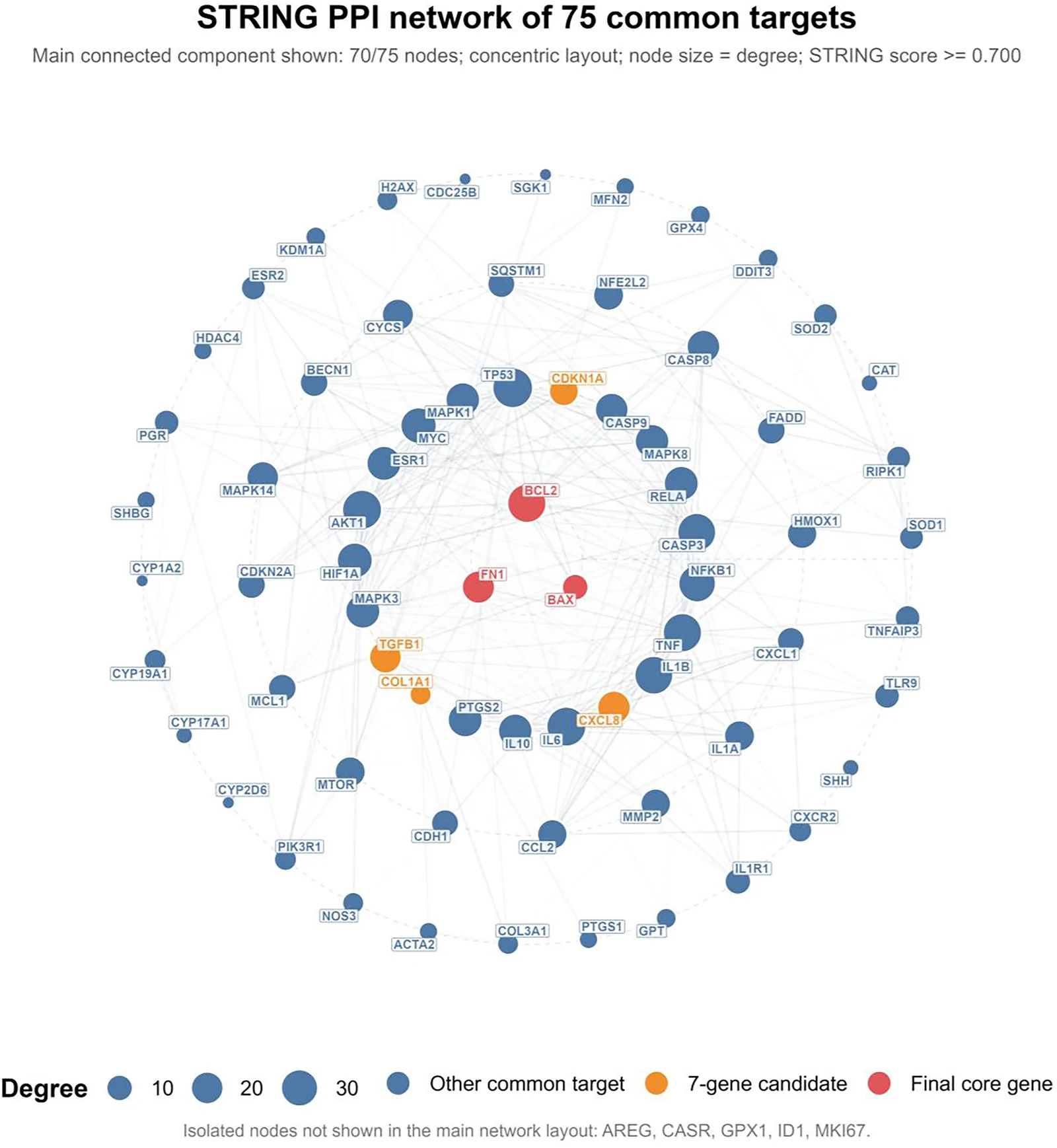
STRING functional-association network of the 75 shared targets. The largest connected component contains 70 of 75 targets. Node size represents degree, and edges indicate high-confidence STRING functional associations (combined score ≥ 0.700). AREG, CASR, GPX1, ID1, and MKI67 are outside the main component.

### 3.3 Functional enrichment analysis

Functional enrichment analysis of the 75 shared targets identified 1,899 significantly enriched GO terms, mainly related to response to reactive oxygen species, oxidative stress response, response to chemical stress, regulation of apoptotic signaling, and gland development. KEGG analysis identified 166 significantly enriched pathways, including AGE-RAGE, TNF, IL-17, apoptosis, and endocrine resistance (**Figure 3**). Complete results are provided in **Supplementary Material 1**, **Supplementary Tables S7**, **S8**.

**Figure 3:**
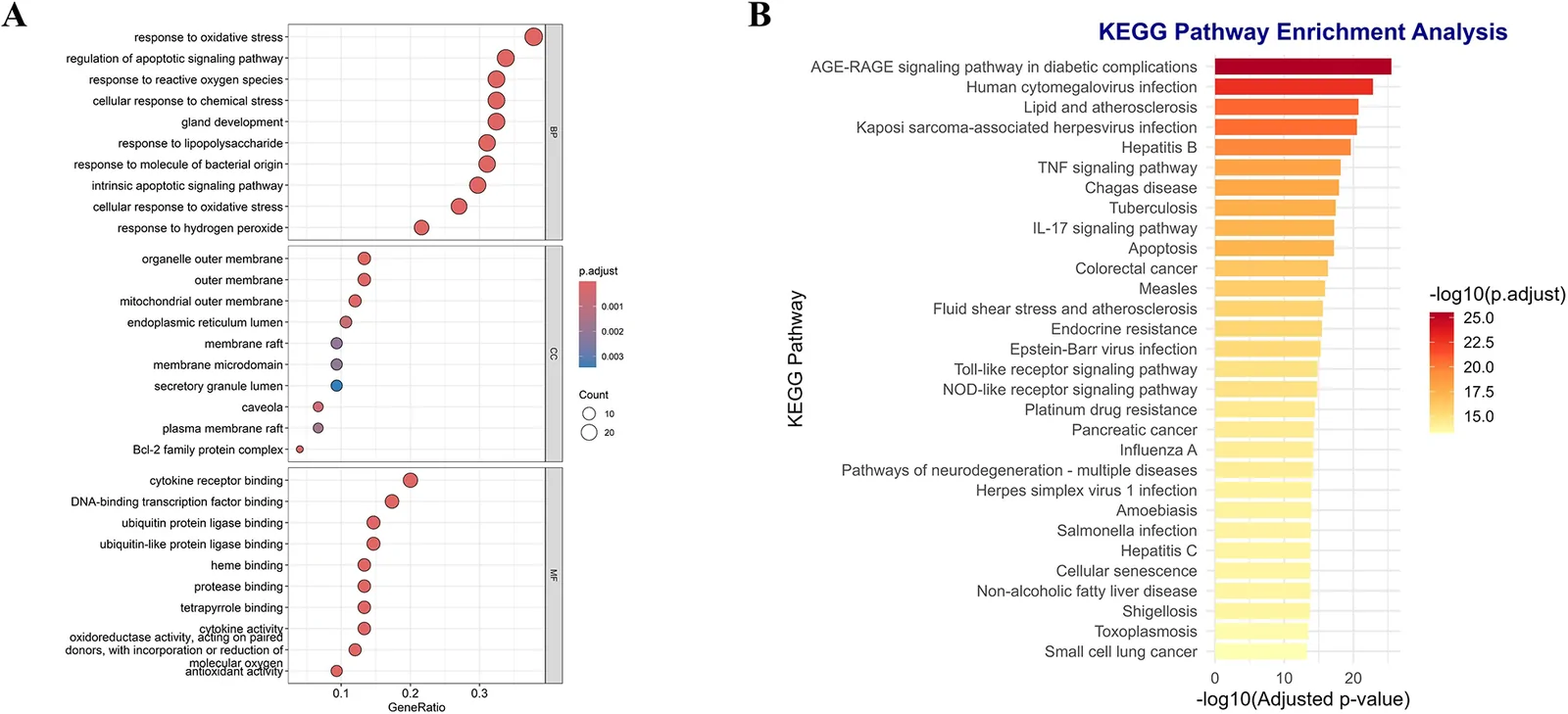
GO and KEGG enrichment analyses of the 75 shared targets. **(A)** GO enrichment analysis. **(B)** KEGG pathway enrichment analysis.

### 3.4 GEO differential expression analysis and identification of candidate genes

In the GSE33630 dataset, GEO2R analysis included 45 normal thyroid tissue samples and 49 PTC tissue samples. Using adj. P.Val < 0.05 and |logFC| > 1, 1,901 differentially expressed probe entries were identified, including 1,060 upregulated and 841 downregulated entries, corresponding to 1,306 unique genes (**Figure 4**). Probe- and gene-level results are provided in **Supplementary Material 1**, **Supplementary Tables S9**, **S10**.

**Figure 4:**
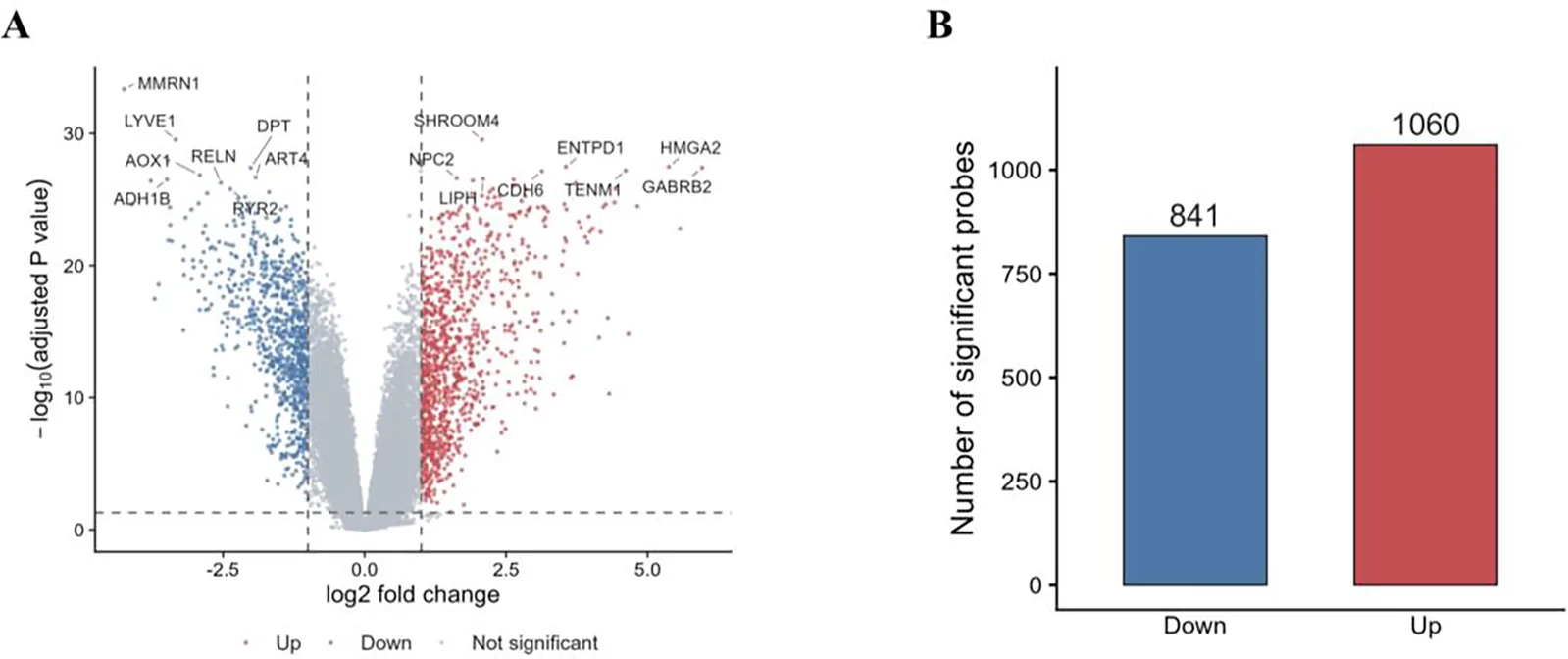
GEO2R differential expression analysis of the GSE33630 dataset. **(A)** Volcano plot. **(B)** Numbers of upregulated and downregulated differentially expressed probe entries.

Intersecting these DEGs with the 75 shared targets yielded seven molecular convergence candidates: BAX, BCL2, CDKN1A, COL1A1, CXCL8, FN1, and TGFB1 (**Figure 1B**; **Supplementary Material 1**, **Supplementary Table S11**).

### 3.5 Identification of core genes using machine learning

Machine learning-based screening was performed using the seven candidate genes. LASSO retained BAX, BCL2, COL1A1, and FN1, whereas SVM-RFE retained BAX, FN1, BCL2, and CXCL8. Their intersection identified BCL2, BAX, and FN1 as the final core genes (**Figure 5**). Across the 50 repeated outer folds, LASSO selection frequencies were 1.00, 0.98, and 1.00 and SVM-RFE frequencies were 0.84, 0.88, and 0.90 for BCL2, BAX, and FN1, respectively. Mean held-out AUCs were 0.970 for L1 logistic regression and 0.966 for linear SVM-RFE (**Supplementary Material 1**, **Supplementary Tables S12**, **S13**).

**Figure 5:**
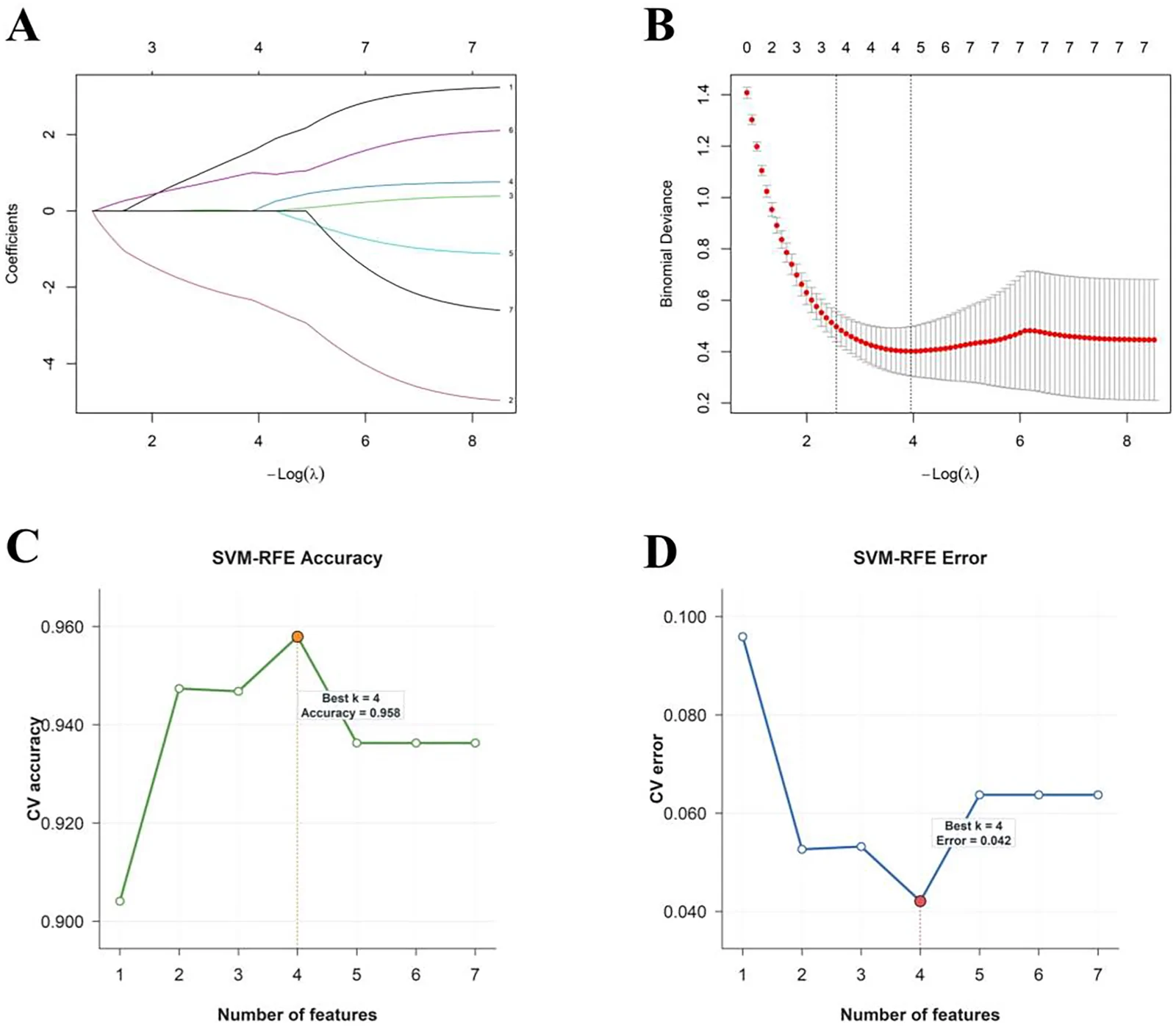
Identification of core genes using LASSO regression and SVM-RFE. **(A)** LASSO coefficient profile. **(B)** Cross-validation curve for LASSO regression. **(C, D)** Feature selection using SVM-RFE.

### 3.6 Expression differences and tumor-normal transcriptomic discrimination of core genes

In the GSE33630 expression matrix, BCL2 was downregulated in tumor tissues (P < 0.001), whereas BAX (P < 0.001) and FN1 (P < 0.001) were upregulated in tumor tissues.

Individual-gene ROC analysis showed tumor-normal AUCs of 0.963 (95% CI: 0.922–0.994) for BCL2, 0.941 (95% CI: 0.888–0.985) for BAX, and 0.965 (95% CI: 0.916–0.996) for FN1 in GSE33630 (**Figure 6**). Corresponding AUCs in GSE60542 were 0.942, 0.907, and 0.955 (**Figure 6**; **Supplementary Material 1**, **Supplementary Table S14**).

**Figure 6:**
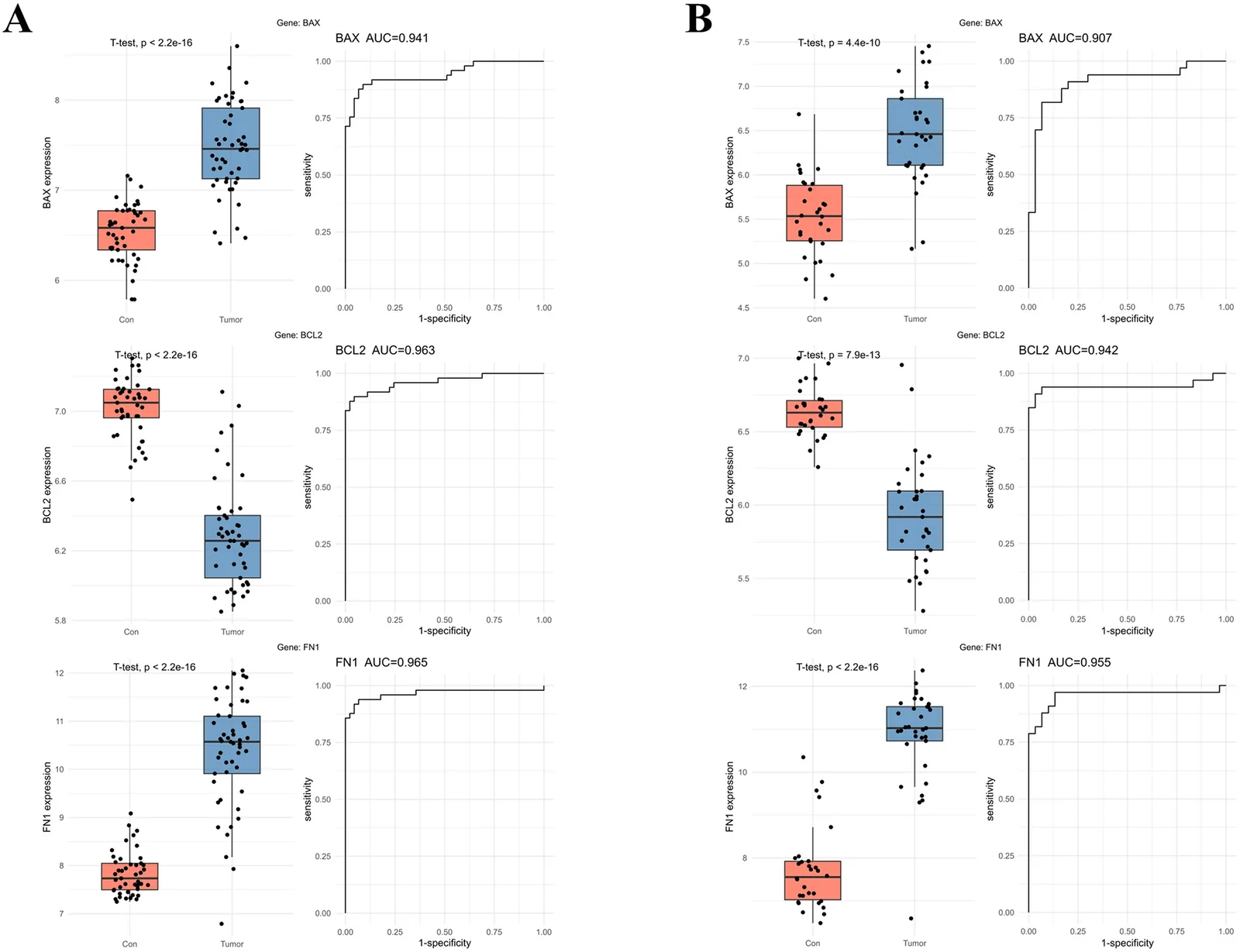
Expression differences and individual-gene tumor-normal ROC curves of the core genes. **(A)** ROC curves in the GSE33630 dataset. **(B)** ROC curves in the GSE60542 dataset.

### 3.7 Public protein expression and immunohistochemical validation

Histological immunohistochemistry images from the Human Protein Atlas showed that the protein expression patterns of the core genes in PTC tissues were generally consistent with the transcriptomic results. BCL2 staining was overall weaker in tumor tissues, whereas BAX and FN1 showed stronger positive staining or broader tissue distribution in tumor tissues. A similar trend was observed in 15 matched samples from clinical settings. All paired t-test and exact Wilcoxon results remained significant after Bonferroni correction. These preliminary tissue-level findings support PTC-associated protein-expression differences (**Figure 7**; **Supplementary Material 1**, **Supplementary Tables S23–S25**).

**Figure 7:**
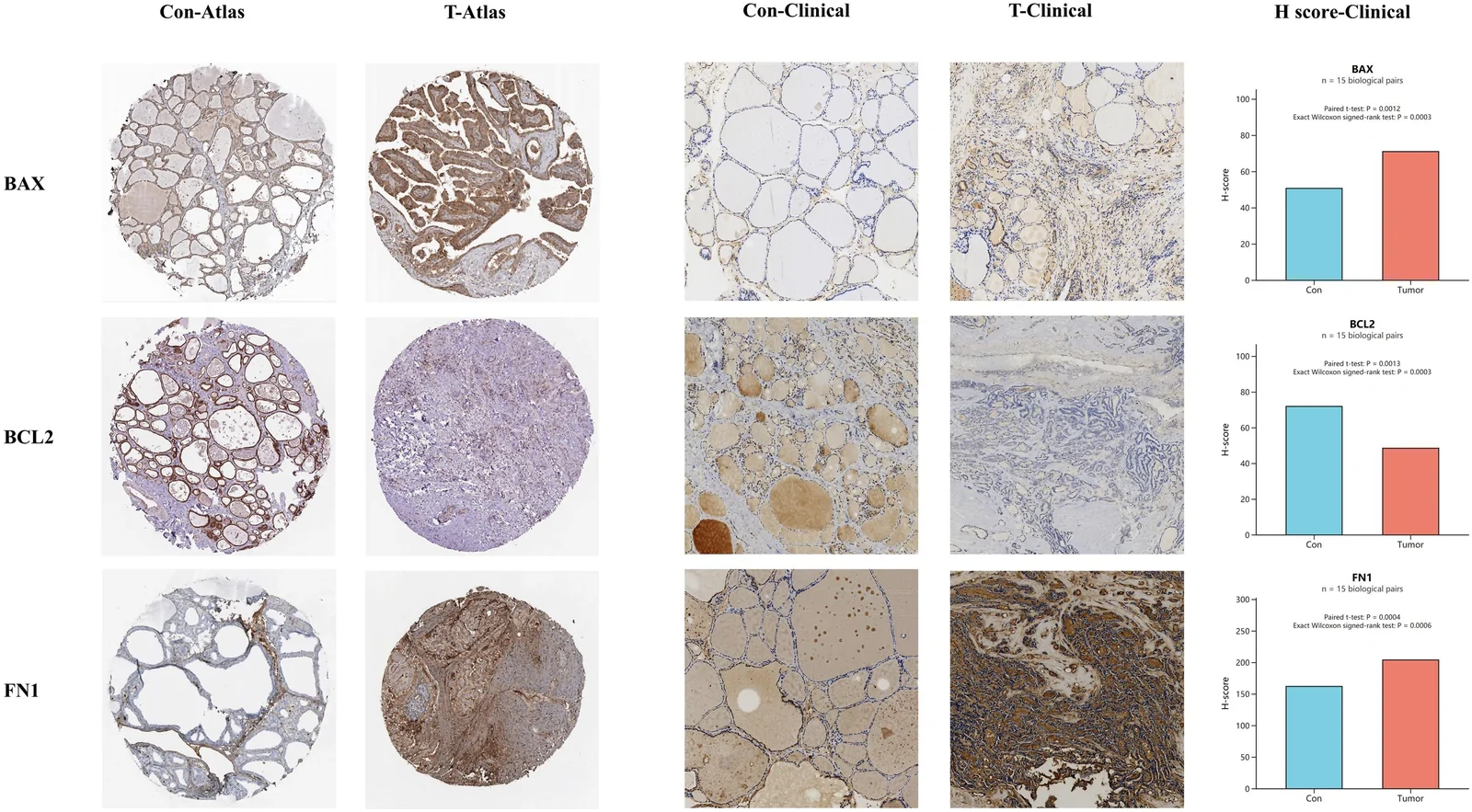
Protein-expression assessment using Human Protein Atlas panels and 15 matched clinical PTC-adjacent tissue pairs. The columns show HPA control and PTC images, clinical adjacent and PTC images, and paired H-scores. The same antibody was used for the two HPA tissue categories within each protein; panel provenance is provided in **Supplementary Material 1**, **Supplementary Table S22**. Clinical characteristics, paired H-scores, and statistical results are provided in **Supplementary Material 1**, **Supplementary Tables S23–S25**. P values displayed in the H-score plots are unadjusted; Bonferroni-adjusted paired-test results are reported in **Supplementary Material 1**, **Supplementary Table S25**. Clinical IHC procedures are described in **Supplementary Material 2**. Clinical images were viewed at a nominal digital magnification of 10× using K-Viewer software.

### 3.8 Immune infiltration differences and core gene correlations

CIBERSORT retained 42 of 94 samples: 15 of 45 normal samples and 27 of 49 PTC samples. Seven estimated immune-cell fractions showed nominal Wilcoxon P < 0.05; after Benjamini–Hochberg correction across 22 comparisons, only the estimated neutrophil fraction remained significant and was higher in PTC (q = 0.0021) (**Figure 8**; **Supplementary Material 1**, **Supplementary Tables S15**, **S16**).

**Figure 8:**
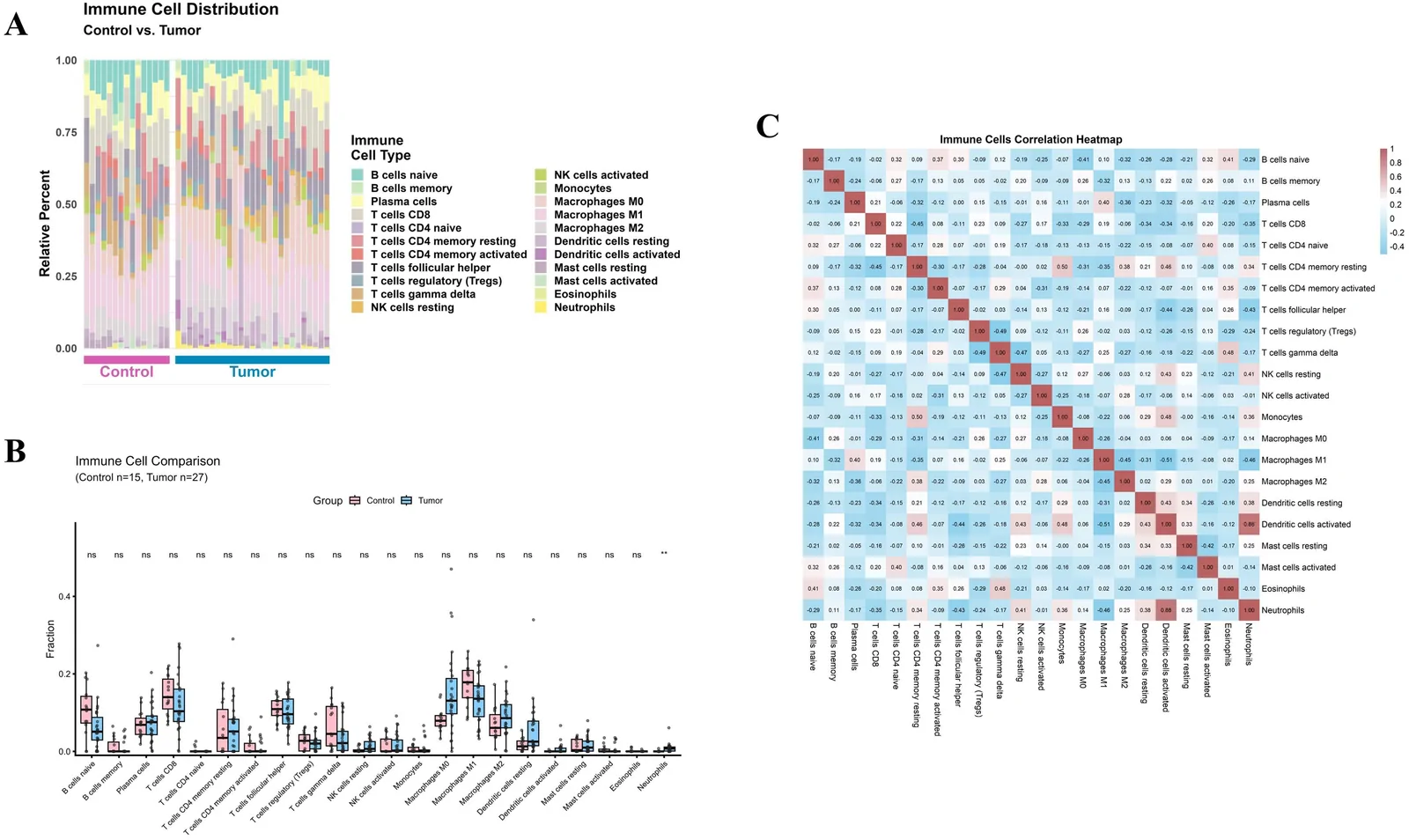
CIBERSORT immune-deconvolution analysis. **(A)** Estimated relative fractions of 22 immune cell types in the 42 retained samples (15 normal and 27 PTC). **(B)** Group comparisons using Wilcoxon rank-sum tests with Benjamini–Hochberg correction across 22 fractions. **(C)** Pearson correlations among estimated immune fractions. *q < 0.05, **q < 0.01, ***q < 0.001.

Across the 42 retained samples, 7 of 66 correlations between core-gene expression and estimated immune-cell fractions remained significant after Benjamini–Hochberg correction, including BCL2–neutrophil (rho = −0.676, q = 5.92 × 10^-5) and BAX–neutrophil (rho = 0.627, q = 0.00029). However, none of the 66 correlations remained significant in the 27 PTC samples analyzed separately (**Figure 9**; **Supplementary Material 1**, **Supplementary Table S17**).

**Figure 9:**
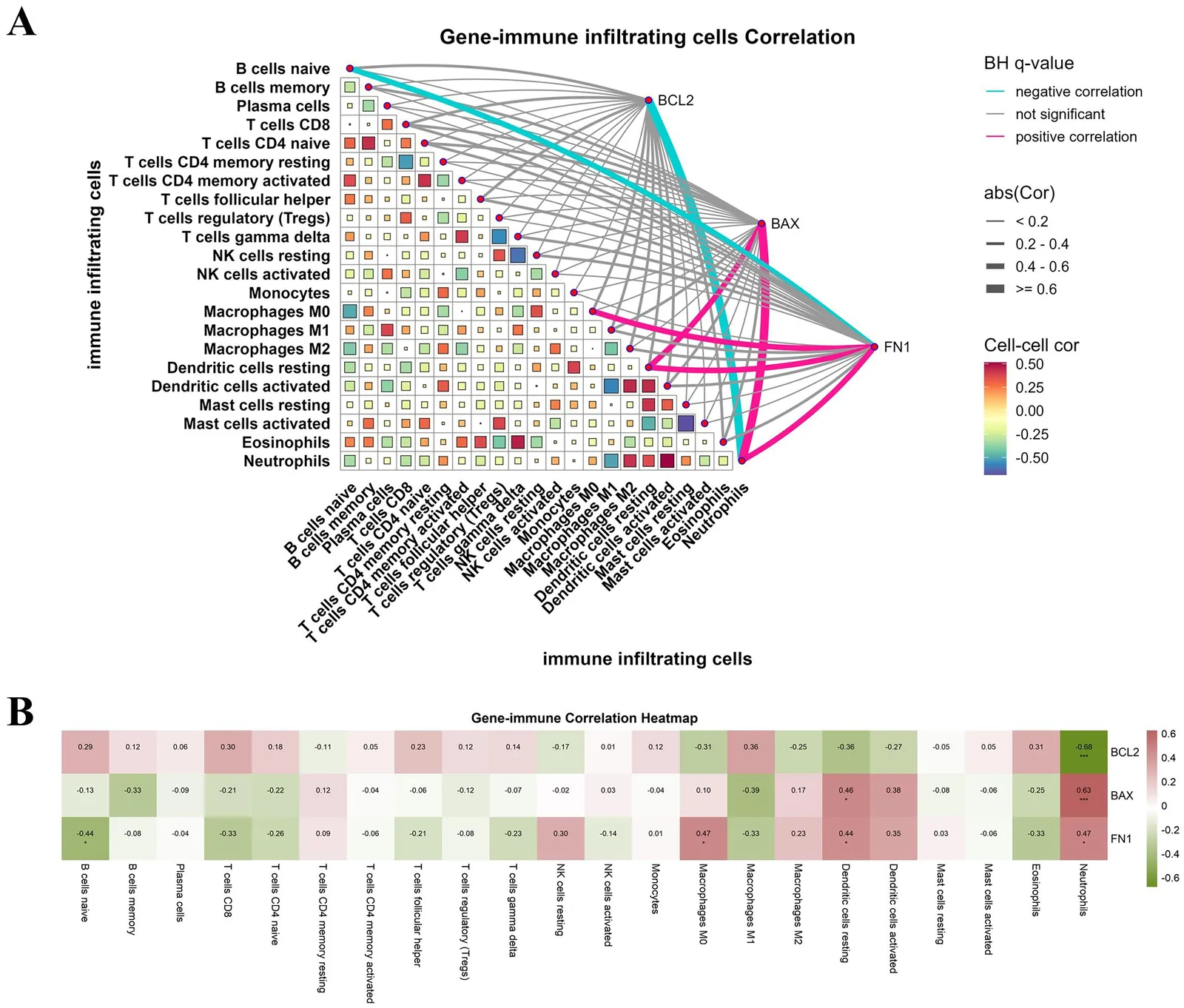
Correlations between core-gene expression and CIBERSORT-estimated immune fractions in the pooled 42 samples. **(A)** Spearman gene–fraction correlations. Pink and turquoise lines indicate positive and negative associations with Benjamini–Hochberg q < 0.05, respectively; gray lines indicate q ≥ 0.05. Line width represents absolute correlation strength. The cell–cell matrix displays descriptive Spearman coefficients. **(B)** Gene–fraction Spearman correlation heatmap. Significance markers are based on Benjamini–Hochberg correction across all 66 gene–fraction tests: *q < 0.05, **q < 0.01, ***q < 0.001. Full pooled and PTC-only results are provided in **Supplementary Material 1**, **Supplementary Table S17**.

### 3.9 Single-cell expression localization of core genes

After quality control, GSE184362 retained 196,724 cells from 23 samples contributed by 11 patients, including 65,745 cells from primary PTC tissues, 50,694 from adjacent non-tumor tissues, 70,249 from lymph-node metastatic lesions, and 10,036 from subcutaneous metastatic lesions. **Figures 10A–F** show UMAP visualizations of tissue groups, annotated cell types, and BAX, BCL2, and FN1 expression in the two datasets. **Figures 10G–I** summarize gene-expression patterns across cell types and tissue groups and compare cellular localization between datasets.

**Figure 10:**
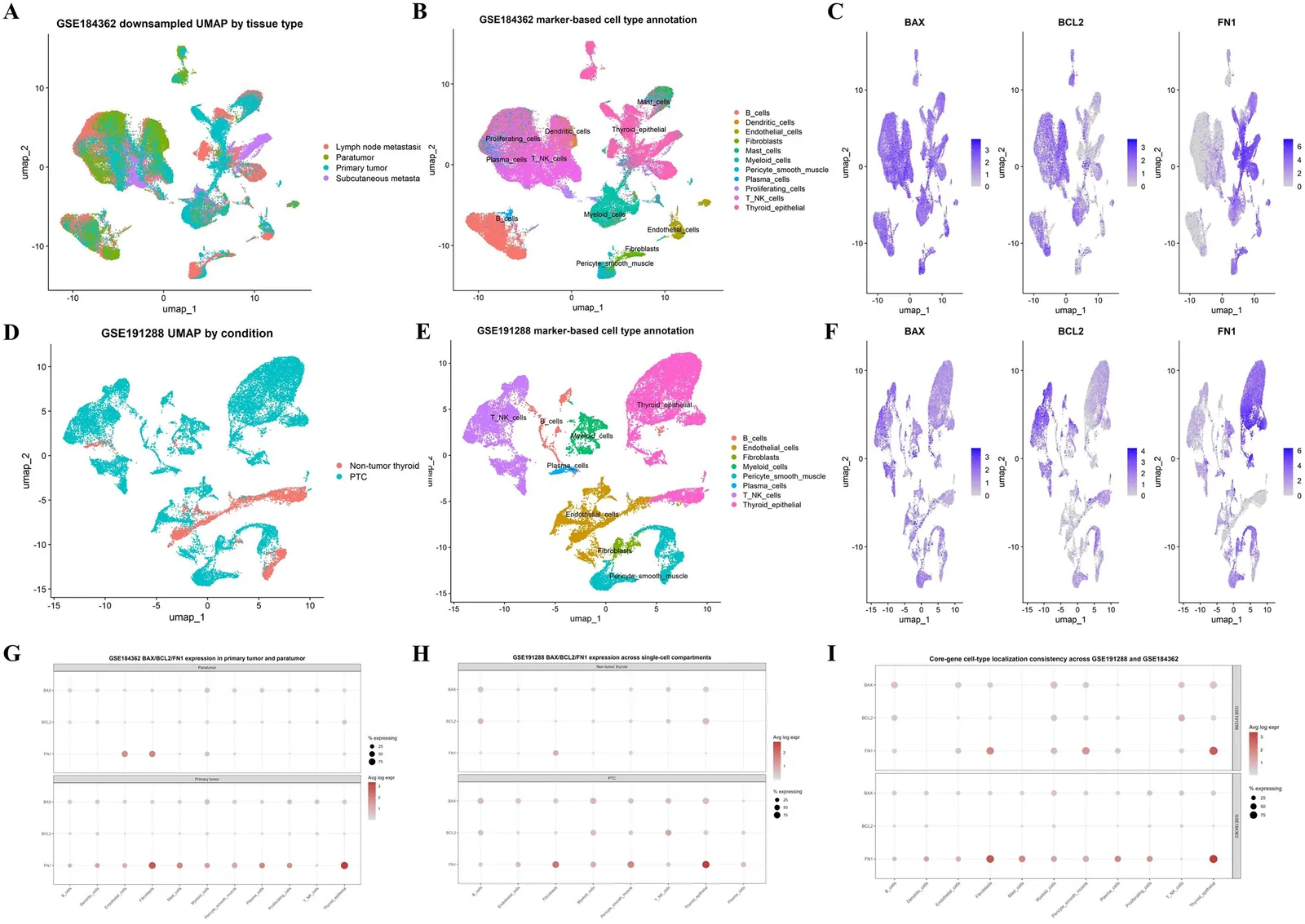
Descriptive single-cell localization of BAX, BCL2, and FN1. **(A–C)** GSE184362 tissue origins, cell-type annotations, and expression distributions. **(D–F)** GSE191288 tissue origins, cell-type annotations, and expression distributions. (**G**, **H**) Expression patterns across cell types and tissue states. **(I)** Cross-dataset localization summary. Dot size indicates the proportion of expressing cells, and color indicates average expression. PTC, papillary thyroid carcinoma; UMAP, uniform manifold approximation and projection.

Cell-type-stratified summaries showed that FN1 signal was prominent in epithelial and stromal compartments and selected immune populations, BCL2 was more prominent in immune-related populations than in thyroid epithelium, and BAX was broadly detected. These descriptive patterns identify plausible cellular contributors to the bulk-expression differences.

GSE191288 retained 31,533 cells and showed broadly similar descriptive cell-type localization (**Figure 10I**; **Supplementary Material 1**, **Supplementary Tables S20**, **S21**).

## 4 Discussion

Thyroid cancer is an endocrine malignancy with a continuously increasing global incidence, and PTC accounts for more than 80% of all thyroid cancer cases. In recent years, microplastics have emerged as environmental contaminants of concern. Their detection in multiple human tissues and biological compartments, including blood, placenta, prostate, and colon, has raised increasing concern regarding their potential health effects (7, 8, 36, 37). But their molecular convergence with thyroid cancer remains insufficiently characterized. In this study, network toxicology and bioinformatics identified 75 shared genes enriched in oxidative stress, inflammatory responses, apoptosis regulation, and extracellular-matrix-related annotations, and BCL2, BAX, and FN1 were further prioritized as core candidates. These findings generate testable hypotheses about convergence between polystyrene-associated annotations and PTC biology.

The findings are broadly consistent with experimental reports concerning polystyrene-related thyroid toxicity. Islam et al. reported thyroid follicular abnormalities and oxidative stress after PS-MPs exposure in mice (38), and Fu et al. reported thyroid follicular-cell pyroptosis after micro/nanoplastic exposure (39). Le et al. used polystyrene nanoplastics in A549 cells (10), whereas thyroid-axis effects have also been studied with nanoparticles in zebrafish (13). However, microplastics, nanoplastics, and soluble polystyrene constituents differ in size, distribution, and biological behavior and should not be treated as interchangeable.

At the level of enriched annotations, the results point to an inflammation–oxidative stress–apoptosis axis within the molecular network shared by polystyrene-associated targets and thyroid cancer. BCL2 and BAX are key regulators of mitochondrial apoptosis (40), and altered expression of the BCL2/BAX axis has been reported in follicular-cell-derived thyroid tumors (41, 42). In the present study, BCL2 was downregulated whereas BAX was upregulated. This concordance supports convergence with established PTC biology.

The identification of FN1 further suggests that extracellular-matrix remodeling may be represented in this convergence. FN1 encodes fibronectin, a protein involved in cell adhesion, migration, matrix deposition, and tumor stromal remodeling. Xia et al. reported that FN1 is highly expressed in PTC with lymph-node metastasis and can promote PTC cell proliferation, migration, and invasion (43). In the present study, FN1 was significantly upregulated in PTC tissues. Its reproducible signal makes it a priority for future experimental validation, while its established role in many cancers limits specificity for polystyrene exposure.

The immune-infiltration and single-cell analyses provide exploratory context for the core candidates. After multiple-testing correction, only the CIBERSORT-estimated neutrophil fraction differed between groups. Although several pooled gene–fraction correlations remained significant, none remained significant within PTC alone. These associations may partly reflect differences in tumor, stromal, or immune-cell composition and do not establish immune-cell recruitment, direct regulation of infiltration by the core genes, or a PS-MPs effect (44, 45).

The value of this study lies in its proposal of a testable candidate molecular framework. BCL2, BAX, and FN1 distinguished established PTC tissue from normal thyroid tissue in two retrospective transcriptomic datasets. However, benign nodules, indeterminate cytology, other thyroid malignancies, and prospective screening populations were not evaluated; these genes therefore cannot currently be considered clinically applicable diagnostic biomarkers. The resampling analysis supports feature stability within the fixed seven-gene panel rather than unbiased end-to-end performance of the complete selection pipeline.

This study has several additional limitations. First, CTD associations and in silico predictions represent different evidence levels, and the broad disease query and GeneCards cutoff may favor well-studied genes, although the source-restriction and threshold analyses retained the three core genes. Second, no patient-level PS-MPs exposure or thyroid particle burden was measured, and no material-characterized PS-MPs exposure experiment was performed in thyroid or PTC cells. Thus, the cross-sectional data cannot establish temporality, dose-response, or causality (46, 47). Third, unequal CIBERSORT sample-retention rates between groups may introduce selection bias. Fourth, the single-cell datasets included few patients, and dedicated doublet detection, cross-sample integration, and donor-level differential-expression modeling were not performed. Finally, the 15-pair IHC cohort was small and retrospective; random selection could not eliminate selection bias, and masking was limited by the recognizable tissue morphology.

## 5 Conclusion

In summary, BCL2, BAX, and FN1 were identified as candidate molecular nodes at the convergence of polystyrene-associated annotations and PTC-associated alterations, with consistent tumor-related expression and preliminary protein-level support. These hypotheses warrant validation in material-characterized exposure experiments and exposure-aware clinical studies.

## Data Availability

The original contributions presented in the study are included in the article/**Supplementary Material**. Further inquiries can be directed to the corresponding author.
