# Supplementary material for: Hypothesis-generating network toxicology analysis of potential molecular convergence between a polystyrene oligomer proxy and papillary thyroid carcinoma
Source: Front Endocrinol (Lausanne). 2026 Oct 14;17:1942165. doi: 10.3389/fendo.2026.1942165 (PMC13713425; doi:10.3389/fendo.2026.1942165)
Supplement: Supplementary file 1 [file Supplementaryfile1.docx]

Clinical Immunohistochemistry and Quality Control

*Polystyrene-proxy and papillary thyroid carcinoma molecular-convergence study*

# 1. Cohort design and tissue definition

The archival laboratory report indicated that the submitted tissues were fixed in 4% paraformaldehyde and paraffin embedded. The clinical IHC analysis was retrospective and exploratory. Fifteen cases were selected by a random-number generator from eligible archived postoperative specimens with pathologic confirmation of papillary thyroid carcinoma (PTC) and available matched tumor and adjacent non-tumor thyroid tissue.

Subacute thyroiditis and other non-Hashimoto inflammatory thyroid diseases were excluded. Hashimoto thyroiditis was not excluded because it may coexist with PTC and could provide clinically relevant contextual information; its presence or absence is reported for every Pair_ID in Supplementary Material 1, Table S23.

Adjacent non-tumor tissue was sampled at least 2 cm from the tumor, showed no grossly evident lesion, and was histologically confirmed to be free of tumor. The term adjacent non-tumor tissue is used because Hashimoto-related changes were not an exclusion criterion. Pathologic TNM categories were recorded according to the AJCC eighth edition: pT1a indicates a tumor ≤1 cm, pT1b >1 to ≤2 cm, and pT2 >2 to ≤4 cm, limited to the thyroid. Based on age and the supplied pT, pN, and M categories, 14 patients were stage I and 1 was stage II.

# 2. Antibodies

| **Marker** | **Primary antibody** | **Study dilution** | **Study-run controls** |
| --- | --- | --- | --- |
| BCL2 | Proteintech  26593-1-AP | 1:400 | Positive and negative controls were included and reviewed before quantification. |
| BAX | Abcam  ab32503 | 1:200 | Positive and negative controls were included and reviewed before quantification. |
| FN1 | Proteintech  15613-1-AP | 1:400 | Positive and negative controls were included and reviewed before quantification. |

# 3. Staining procedure

• Deparaffinization and rehydration: xylene I for 12 min, xylene II for 12 min, absolute ethanol for 6 min, 95% ethanol for 6 min, 85% ethanol for 6 min, then running water for 2 min.

• Antigen retrieval: Tris-EDTA buffer (pH 9.0) in a microwave oven (medium power for 8 min to boiling, 8 min without heating, then medium-low power for 7 min), followed by natural cooling and PBS washes three times for 5 min each.

• Endogenous peroxidase blocking: 3% hydrogen peroxide for 25 min at room temperature in the dark, followed by three PBS washes.

• Blocking: 3% bovine serum albumin for at least 30 min at room temperature.

• Primary antibody incubation: overnight at 4°C using the marker-specific antibodies and dilutions shown above.

• Detection: POLY-HRP goat anti-rabbit secondary antibody (Hangzhou Haoke Biotechnology, HKI0026) for 50 min at room temperature, followed by three PBS washes.

• Chromogen and counterstain: freshly prepared DAB, with development monitored microscopically and stopped with water; hematoxylin counterstaining for approximately 2-3 min, differentiation for 2 s, and bluing for 15-30 s.

• Dehydration, clearing, mounting, microscopic inspection, and image acquisition were completed according to the laboratory SOP.

# 4. Controls, masking, and quality control

## 4.1 Run controls

Positive and negative controls were included in each staining run and were inspected before H-score quantification; only runs with acceptable control staining were analyzed.

## 4.2 Masking

H-score quantification was performed by professional personnel independent of the study authors and without conflicts of interest. During scoring, the assessor was masked to the original specimen and medical-record identifiers and to all patient-level clinicopathologic information. Complete masking to tissue category was not feasible because PTC and adjacent non-tumor thyroid tissue could be morphologically distinguishable. After scoring, results were linked to clinical information through de-identified Pair_IDs.

## 4.3 Slide and image-analysis QC

Samples were processed under the laboratory's pathology SOP, and only slides that passed post-staining microscopic inspection were included. Whole-tissue regions were selected in Visiopharm for target-object quantification, thereby avoiding selective high-power-field sampling.

# 5. H-score calculation and statistical analysis

Cells were classified as negative (0), weakly positive (1+; pale yellow), moderately positive (2+; brown-yellow), or strongly positive (3+; brown). The H-score was calculated over the whole selected tissue region as: H-score = 1 × percentage of 1+ cells + 2 × percentage of 2+ cells + 3 × percentage of 3+ cells, yielding a range from 0 to 300. Positivity was defined as (1+ + 2+ + 3+ cells) / total cells.

Each marker was analyzed in 15 matched tumor-adjacent biological pairs. Raw Pair_ID-linked values and full statistics are reported in Supplementary Material 1, Tables S24 and S25.
